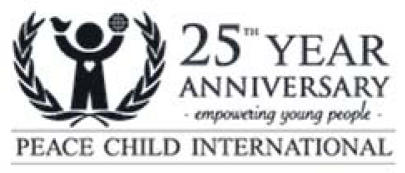# EHPnet: Peace Child International

**Published:** 2007-10

**Authors:** Erin E. Dooley

Peace Child International (PCI) works through affiliated groups in more than 100 countries to inspire young people to become more socially and environmentally conscious through education, leadership development, and community involvement. PCI works with the UN and its agencies to promote its mission and programs, described at **http://www.peacechild.org/**.

The PCI program “Be the Change” funds small community action projects developed and implemented primarily by people under age 25 in support of the eight UN Millennium Development Goals adopted in 2000. Recent projects have set up a bicycle taxi service in Kenya, launched a water and sanitation effort in Uganda, and organized an eco-library in Armenia.

PCI is currently compiling a children’s book about environmentally friendly transportation around the world, which will be distributed to London primary schools. Draft spreads for the book and calls for contributions are available on the website. The site also offers issues of *TUNZA*, UNEP’s quarterly magazine for young people, through its Resources section. Each issue focuses on themes such as green cities, recycling, and food and the environment, with numerous articles as well as a section where UNEP experts answer questions sent in by readers.

The site also hosts information on the biennial World Youth Congresses that PCI began organizing in the late 1990s. These conferences convene youth from around the world to recognize their work in furthering sustainable development practices as outlined in the Millennium Development Goals. The next congress is scheduled for Québec in August 2008.

## Figures and Tables

**Figure:**